# FACTORS ASSOCIATED WITH OBJECTIVELY MEASURED LIFE-SPACE AND GREEN SPACE IN AGING ADULTS

**DOI:** 10.1093/geroni/igae098.3975

**Published:** 2024-12-31

**Authors:** Michelle Odden, Annabel Tan, Sylvie Dobrota Lai, Sheila Mwanda, David Rhodes, Suzanne Judd, Vishnu Ravi

**Affiliations:** Stanford University, Palo Alto, California, United States; Stanford University, Palo Alto, California, United States; Stanford University, Palo Alto, California, United States; Stanford University, Palo Alto, California, United States; University of Alabama Birmingham, Birmingham, Alabama, United States; University of Alabama Birmingham, Birmingham, Alabama, United States; Stanford University, Palo Alto, California, United States

## Abstract

Life-space is an important aging health outcome, and measurement of the life space allows for a better characterization of participants’ environments than their home address. We developed a mobile phone application to passively capture life-space in older adults and sampled Google street view images from participants’ life-space to characterize the environments where they spent their time. We recruited 82 participants aged 65 and older from the REasons for Geographic And Racial Differences in Stroke (REGARDS) study. They were followed for 2 weeks and invited to complete nightly mobile questionnaires. We measured features of the built environment that capture walkability (sidewalks, benches, streetlights), neighborhoods (houses), and green space. Among 82 participants, 76 completed all 14 days of data collection. Median daily maximum distance from home was 6.6 km (IQR: 1.3 to 15.5, range: 0 to 5074.9 km). Median distance traveled over two weeks was 477.1 km (IQR: 234.4, 817.13; range: 39.1 to 31,743.1 km). Adults younger than 70 years, women, Black participants, and those with income >$75,000 per year had larger life-space and traveled further from home. Engagement in exercise, number of chronic conditions, and depressive symptoms were not associated with life-space metrics. Participants with 2 or more chronic conditions spent less time in green space, although the effect sizes did not reach statistical significance after multivariable adjustment (standardized effect sizes -0.61 (95% CI: -1.35, 0.14) and -0.05 (95% CI:-0.11, 0.01), respectively). In summary, there was wide variation in objectively-measured life-space and we found sociodemographic patterning of life-space movement.

